# Implantation of silicon dioxide-based nanocrystalline hydroxyapatite and pure phase beta-tricalciumphosphate bone substitute granules in caprine muscle tissue does not induce new bone formation

**DOI:** 10.1186/1746-160X-9-1

**Published:** 2013-01-04

**Authors:** Shahram Ghanaati, Samuel E Udeabor, Mike Barbeck, Ines Willershausen, Oliver Kuenzel, Robert A Sader, C James Kirkpatrick

**Affiliations:** 1 Institute of Pathology, REPAIR-Lab, Johannes Gutenberg University Mainz, Langenbeckstrasse 1, Mainz, 55101, Germany; 2 Department for Oral, Cranio-Maxillofacial and Facial Plastic Surgery, Medical Center of the Goethe University Frankfurt, Frankfurt am Main, Germany; 3 Department for Operative Dentistry, Johannes Gutenberg University Mainz, Mainz, Germany

**Keywords:** Osteoinduction, Cerasorb, NanoBone, Nanocrystalline, ß-tricalciumphosphate, Hydroxyapatite, Ectopic bone formation

## Abstract

**Background:**

Osteoinductive bone substitutes are defined by their ability to induce new bone formation even at heterotopic implantation sites. The present study was designed to analyze the potential osteoinductivity of two different bone substitute materials in caprine muscle tissue.

**Materials and methods:**

One gram each of either a porous beta-tricalcium phosphate (β-TCP) or an hydroxyapatite/silicon dioxide (HA/SiO_2_)-based nanocrystalline bone substitute material was implanted in several muscle pouches of goats. The biomaterials were explanted at 29, 91 and 181 days after implantation. Conventional histology and special histochemical stains were performed to detect osteoblast precursor cells as well as mineralized and unmineralized bone matrix.

**Results:**

Both materials underwent cellular degradation in which tartrate-resistant acid phosphatase (TRAP)-positive osteoclast-like cells and TRAP-negative multinucleated giant cells were involved. The ß-TCP was completely resorbed within the observation period, whereas some granules of the HA-groups were still detectable after 180 days. Neither osteoblasts, osteoblast precursor cells nor extracellular bone matrix were found within the implantation bed of any of the analyzed biomaterials at any of the observed time points.

**Conclusions:**

This study showed that ß-TCP underwent a faster degradation than the HA-based material. The lack of osteoinductivity for both materials might be due to their granular shape, as osteoinductivity in goat muscle has been mainly attributed to cylindrical or disc-shaped bone substitute materials. This hypothesis however requires further investigation to systematically analyze various materials with comparable characteristics in the same experimental setting.

## Introduction

The search continues for an “ideal” bone substitute for the support, augmentation or replacement of bony tissue defects. Among other properties, these bone substitutes should ideally possess osteoconductivity, osteoinductivity and osteogenicity. Despite the increase in the number of surgical procedures that require bone grafts, there is still no ideal bone graft substitute [1]. Although autografts are the gold standard that all other alternatives must meet or exceed, they have significant limitations, including donor site morbidity, inadequate tissue quantity, inappropriate forms [2,3] and sometimes the need for general anesthesia for their harvest [4,5]. These limitations have prompted increasing interest in alternative bone grafts. Allografts may be cancellous, cortical or a combination of these. Though they are attractive sources, several problems arise when using them, including the risk of disease transmission, immunogenicity [6] loss of biological and mechanical properties secondary to processing, increased cost, and lack of availability due to financial and religious-cultural concerns [1].

The drawbacks associated with natural bone grafts have led to the production of a large number of synthetic grafts. The latter are readily available, do not cause an antigenic response and can easily be tailored to the intended application. However, the biological performance of synthetic bone grafts in terms of initiation and support of bone growth is inferior to natural bone grafts [7]. Their biological behaviour depends upon their chemical composition and physicochemical structure.^4^ A group of these synthetic biomaterials are termed osteoinductive biomaterials. These materials are potentially “intelligent” bone graft substitutes in that they are able to induce the *in vivo* environment to form bone [7]. This also refers to their ability to stimulate and support the proliferation and differentiation of mesenchymal progenitor cells of the host tissue [8] when implanted in ectopic (i.e., extraskeletal) sites, together with the induction of bone formation [9,10]. Although the exact process of osteoinduction by biomaterials is still largely unknown, studies have shown that biomaterials need to meet very specific requirements in terms of macrostructure, microstructure and chemical composition in order to be osteoinductive [7,11].

The osteoinductive potential of NanoBone*®* (NB) hydroxyapatite/silicon dioxide (HA/SiO_2_)-based nanocrystalline bone substitute has been demonstrated in an *in vivo* study in mini pigs by Götz *et al.*[12]. The authors reported both new bone formation and osteogenic differentiation which they claimed were better in the subcutaneous tissue than in the intramuscular implantation sites.

Our group has also been able to demonstrate in clinical trials the cellular pathway involved in NB degradation [13] and its osteoconductive capacity to promote sufficient new bone formation required for stable implant placement after three months [14].

The main aim of the present study was to investigate the osteoinductive potential of NB granules in goat intramuscular implantation sites in comparison with *Cerasorb®* a pure-phase beta tricalciumphosphate (ß-TCP) granules.

## Materials and methods

### Bone grafting substitute NanoBone®

NanoBone® (Artoss, Rostock, Germany), a fully synthetic bone substitute granule, consists of hydroxyapatite crystallites with an average size of 60 nm in each crystallographic direction that are embedded in a matrix of silica gel. It is produced by a sol–gel-technique at temperatures below 700°C, avoiding sintering of the nanocrystalline hydroxyapatite [15]. In the transition process from sol to gel, a loose connection of hydroxyapatite crystals with the SiO_2_ molecules takes place. This connection is responsible for a nanoporous structured bone substitute. The biomaterial is characterized by numerous open bonds, which are responsible for an internal surface of up to 84 m^2^/g in size. The pore size distribution within the silica gel ranges from 10 to 20 nm in diameter. Macroscopically, the fir cone-shaped NanoBone® granules possess an average length of 2 mm and an average diameter of 0.6 mm with a porosity of 60% - 80%.

### Bone grafting substitute cerasorb®

The details of the synthesis of pure phase β-TCP and the fabrication of Cerasorb® M (Curasan, Kleinostheim, Germany) are described elsewhere [16]. Briefly, pure phase β-tricalcium phosphate was synthesized by a solid-state reaction. After crushing and sieving a portion (< 63 μm) of the generated material, the ceramic particles were mixed with an organic porogen and pressed to rods. During a second sintering step (≥ 1000°C) the porogen disappeared. The resulting ceramic was highly porous with macropores of about 50–500 μm interconnected by micropores. After crushing the porous rods to splint granulates, the desired granulate sizes were reached by sieving. Finally Cerasorb® M was sterilized by gamma irradiation.

### Experimental design of the muscle model in goats

This study was performed in an accredited laboratory (RCC Ltd, Zelgliweg 1, 4452 Itlingen/Schwitzerland) in accordance with the Swiss Animal Protection Law under license (no. BL338) and by following internationally recognised guidelines. Six female goats (Capra hircus, Olsberg, Switzerland) were kept in agricultural animal husbandry in group housing of 100 square meters for six animals. Straw bedding was provided. Standard goat maintenance diet (Landi Jungfrau AG, Switzerland) was presented twice daily with water *ad libitum*. The animals were allocated into three groups of two animals each for the following time points: 28, 91 and 181 days.

Bone induction in ectopic tissue was analyzed by implanting the biomaterial in muscle pouches of the right Musculus longissimus dorsi (M. long. dorsi) and Musculus biceps femoris (M. biceps femoris). In each animal, one muscle pouch was operated without biomaterial implantation for the above mentioned time points (sham operation). These empty muscle pouches were used to classify the inflammatory response related to the operation in the absence of biomaterial implantation.

After medication with propofol at a dose sufficient to ensure appropriate induction of anesthesia (5–7 mg/kg i.v.), the level of anesthesia was maintained by means of isoflurane/oxygen via face-mask. Prior to surgery, the respective sites were shaved and disinfected with a standard of iodine/povidone (Betadine®) solution. Four to five muscle pouches per animal, either in the M. longissimus dorsi or M. biceps femoris, were formed after skin incision and blunt preparation with surgical scissors of approximately 0.5 cm diameter. A small amount of the biomaterial, i.e. 1.0 g, was deployed using glass-weighing boats with spouts for reliable positioning of the granular material in the muscle pouches. The muscle pouches were then closed with a button seam, likewise the following layers and the skin. All work was performed under sterile conditions. Postoperative analgesia consisted of single intramuscular injections of Metamizol at doses of 20 mg/kg body weight. During the acclimatization and post-surgical observation periods, the animals were only transiently separated for the assessment of clinical signs and body weights.

### Tissue preparation and histology for the muscle model

The animals were anesthetized with a captive bolt pistol and sacrificed by exsanguination. Immediately after death, the implantation beds containing the biomaterials were explanted together with the surrounding muscle pouches and fixed in 4% formalin for 24 hours for further histological and immunohistochemical analysis. The implant sites of each of the six animals were cut into three segments of identical dimensions of 4 mm thickness according to previously described methods.^16^ The central segment of the intramuscular pocket containing the biomaterial was used to identify osteoclast-like cells by staining for tartrate-resistant acid phosphatase (TRAP) and for the identification of osteoid and/or bone matrix by Movat´s pentachrome and Sirius red staining according to previously described methods [13,17,18].

## Results

### Histological results in goat muscle

The tissue reactions to the granules in goat muscles were varied and are highlighted under separate headings below.

### Tissue reaction to nanocrystalline hydroxyapatite granules

**Figure 1:**
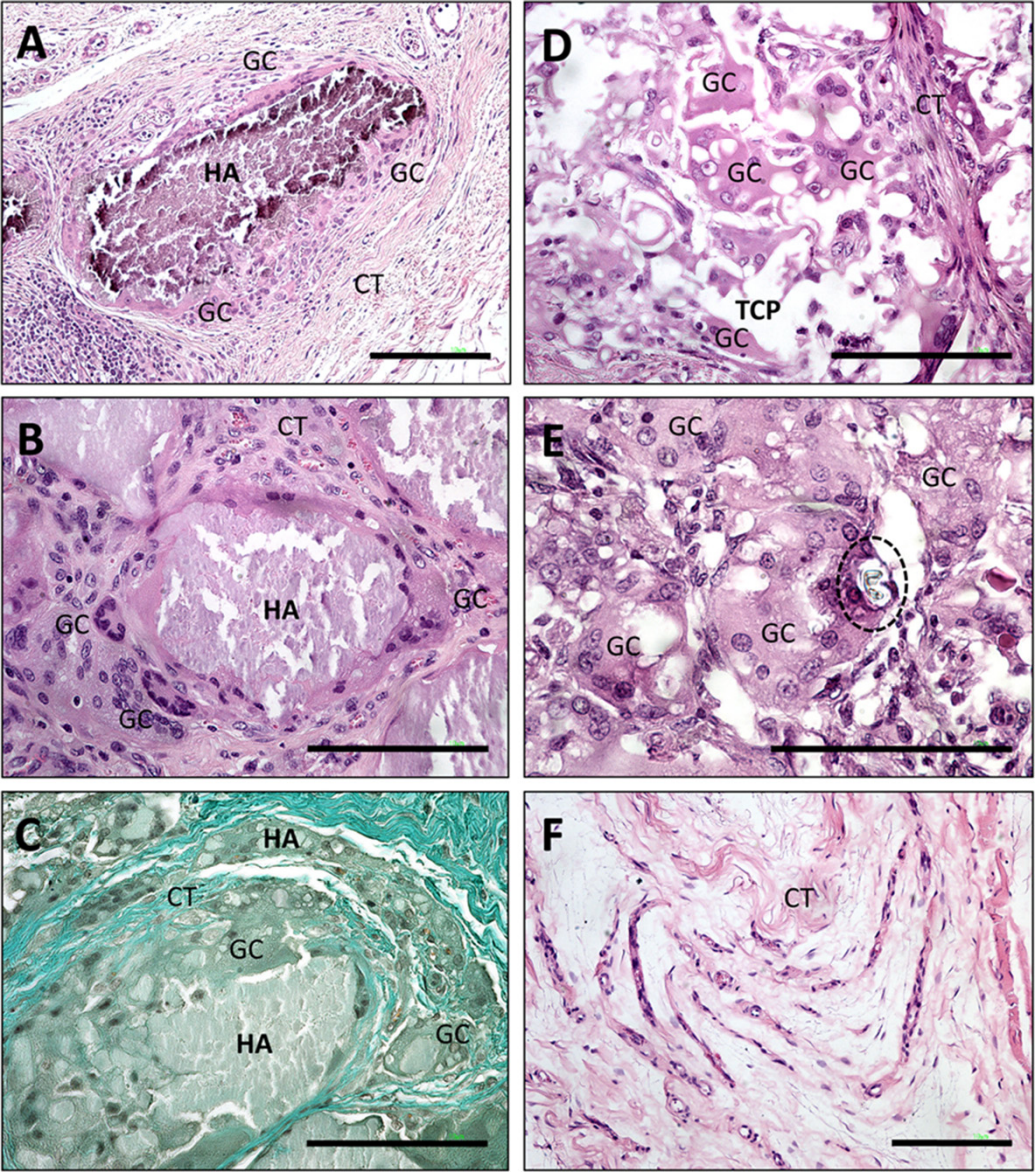
**Shows images of the tissue reaction to the HA- and the β-TCP-based bone substitutes within goat muscle at given time points of the study (day 28–181).** A-C) display the tissue reaction to the HA-based nanocrystalline biomaterial on day 28 (**A**), day 91 (**B**) and day 181 (**C**) after implantation respectively. Note the osteoclast-like giant cells (GC) in close contact with the material (HA) on day 28 and day 91, with no sign of biomaterial breakdown (A: H&E-staining, 100x magnification, scale bar = 10 μm, B: H&E-staining, 200x magnification, scale bar = 10 μm), CT=connective tissue). On day 181 (C) a breakdown of granule integrity by fibrous tissue and phagocytic cells is observed, which resulted in many small particles within the implantation bed (Masson-Goldner-staining, 100x magnification, scale bar = 10 μm). D-F) show the tissue reaction to the β-TCP-based material on day 28 (**D**), day 91 (**E**) and day 181 (**F**). On day 28 the material (TCP) is surrounded and invaded by many multinucleated giant cells (GC) (H&E-staining, 200x magnification, scale bar = 10 μm). On day 91 only few remnants of the biomaterial can be detected, while osteoclast-like giant cells (GC) dominate the implant side. Fragments of the bone substitute are detectable in the cytoplasm of the multinucleated giant cells (dashed line) (H&E-staining, 400x magnification, scale bar = 10 μm. On day 181 fibrous tissue remains after the fast degradation of the biomaterial (H&E-staining, 100x magnification, scale bar = 10 μm).

**Figure 2:**
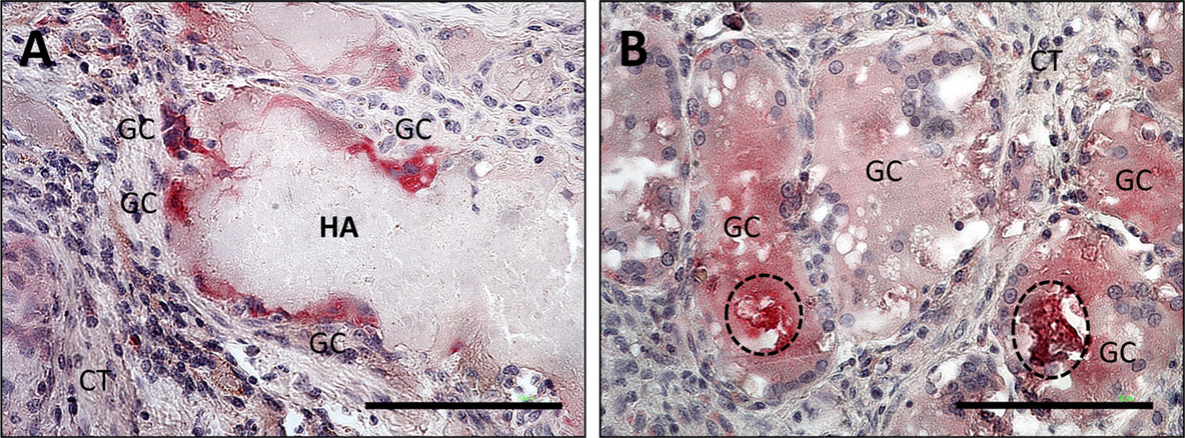
**Shows comparative TRAP-staining of the HA-based (A) and the β-TCP-based (B) bone substitutes.** The HA-based material only induces the formation of multinucleated giant cells (GC) with few nuclei on its surface, while the β-TCP-based material induces the fusion of voluminous multinucleated giant cells that contain particles of the biomaterial within their cytoplasm (dashed line) (A+B: TRAP-staining, 400x magnification).

Within the implantation bed of the nanocrystalline hydroxyapatite, the surface of the granules was surrounded by multinucleated giant cells. The degradation of the biomaterial was initiated from the periphery of the material and continued for the duration of the study (Figure 1A-C). Accordingly, the size of the granules decreased from day 29 until day 181 after implantation (Figure 1A-C). The multinucleated giant cells within the implantation bed of this bone substitute were mostly TRAP-negative. However, a few TRAP-positive multinucleated giant cells were also located on the surface of the HA-based material (Figure 2A). At day 91, the HA granules were well integrated within the implantation bed. At day 181 after implantation, the granules were penetrated by fibrous tissue and phagocytic cells resulting in their breakdown and disintegration (Figure 2C). At no time within the observational period osteoblasts or bone-specific matrix could be identified via immunohistochemical staining methods in any implantation bed of this nanocrystalline bone substitute.

### Tissue reaction to the β-TCP granules

Within the implantation bed of the β-TCP-based bone substitute, all granules were well integrated. As early as day 28 after implantation, connective tissue fibers as well as phagocytic cells, such as macrophages and multinucleated giant cells, penetrated the center of the granules. Also at this time point, a progressive degradation of the material was visible, and small particles of the granules were incorporated into multinucleated giant cells (Figure 1D). The β-TCP-based granules were almost completely degraded at day 91 after implantation (Figure 1E). At this time point, the major part of the implantation bed was invaded by multinucleated giant cells, which contained small components of the granules within their cytosol. At day 181 after implantation, the β-TCP-based bone substitute material was totally degraded and only collagenous fibrous tissue with a few vessels remained (Figure 1F). Within the implantation bed, TRAP-positive multinucleated giant cells that were involved in the degradation of the biomaterial could be found. However, their number seemed to be higher within the implantation bed of the β-TCP-based bone substitute when compared with the implantation bed of the nanocrystalline material (Figure 2B). Neither osteoblasts nor bone-specific matrix was found in any of the implantation beds of this β-TCP-based bone substitute at any time point.

## Discussion

The present study demonstrated that the two bone substitute materials showed differences in the extent of multinucleated giant cell formation within their implantation beds. Multinucleated giant cells in the implantation bed of the β-TCP-based bone substitute were mostly TRAP-positive, whereas only a few TRAP-positive giant cells were located on the surface of NB. The higher presence of multinucleated giant cells, especially the TRAP-positive subpopulation within the implantation bed of β-TCP, reflects the influence of the chemical composition of this bone substitute on the expression of the degrading enzyme tartrate-resistant acid phosphatase [19,20]. Accordingly, the high presence of multinucleated giant cells might be related to the composition of the used β-TCP and might be, in addition to dissolution, a reason for its comparably faster degradation [19,20]. Multinucleated giant cells are known to originate from mononuclear phagocytic cells such as macrophages [21-23] and their presence reflects the foreign body reaction, which is described for such biomaterials [13,20,24]. Thus, gene expression of degrading enzymes like TRAP is dependent on the characteristics of the biomaterial [21,22,25].

The degradation pattern which was observed for NB in this study is similar to what our group had previously reported following material implantation in subcutaneous tissue of Wistar rats [24]. This material underwent a more continuous degradation over time, and the breakdown of the granules into particles took place at later stages of the study.

The control over the degradation rate of a biomaterial is an essential aspect for its contribution to bone remodeling. Studies investigating the osteoinductive properties of macroporous calcium phosphate cements postulated that fast biomaterial degradation may have a negative influence on its osteoinductive characteristics [26]. Thus, a fast degradation will result in a connective tissue influx, which might inhibit bone regeneration in the respective defect [26]. However, it remains unclear to what extent this connective tissue influx into a bone defect as a result of biomaterial degradation might undergo differentiation into bone over time. Therefore, the activity of degrading cells could be controlled by the physicochemical characteristics of the material, as shown by the two analyzed materials.

In this study, we have studied the potential osteoinductivity of the two bone substitute materials by means of histological and histochemical staining methods. Throughout the study period, no osteoblasts or bone-specific matrices could be found in any implantation bed of the used NB granules. These data are in accordance with previous studies, which also failed to show osteoinductive properties of HA-based bone substitutes within goat muscle when compared to various forms of porous biphasic calcium phosphates (BCP) [11]. However, it must be mentioned that the other authors used cylindrically-shaped calcium phosphate ceramics [11] and not granules as in the present study. Despite the presence of micro- and nanopores, NB in its granular form within caprine muscle probably did not induce sufficient mineral ion influx and protein-related surface modifications, which are suggested as a requirement to trigger osteoinduction [27-29]. On the other hand, another *in vivo* study in mini pigs reported a marked osteoinduction within subcutaneous as well as muscle implantation sites induced by the very same NB-granules [12]. Thus, we assume that the lack of osteoinductivity of NB in the present study could be explained on the basis of its application form, i.e. granules and the animal species.

It is noteworthy that β-TCP granules, which were used as controls in the present study, stimulated neither osteoblasts nor bone-specific matrices in any of the implantation beds throughout the observational period. The failure of β-TCP to induce ectopic bone formation when applied as a single bone substitute material has also been previously described [30]. In combination with bone marrow stromal cells [31], however, and along with hydroxyapatite as biphasic calcium phosphate ceramics [32-34], it has been shown to induce different degrees of osteoinductivity, even in ectopic tissues. With regard to objectivity, it has to be considered whether the used ß-TCP granules would have shown some osteoinductive properties, when implanted into the subcutaneous or muscle tissue of the mini-pig as was done for NB [12]. Furthermore, it has to be emphasized that for ß-TCP, osteoinduction has been demonstrated in dogs [35,36]. However, the materials used for the dog study were of different morphology, namely either cylindrical or disc shape.

The reasons for lack of ectopic bone formation in the present study are not apparent. Despite the fact that the principle behind the process of osteoinduction is largely unknown, it is believed to be positively influenced by the chemical composition of biomaterials [37], their sintering temperature [38], material dissolution [37], macro- and microporosity [36-38], implant size and the applied animal model [34,39]. Besides these factors, inflammation itself might be an influencing factor for bone induction. The release of cytokines, which consequently lead to a higher circulation within the implantation bed, stimulate circulating stem cells to differentiate into osteoblastic cells [34]. Accordingly, a better understanding of the degradation-related inflammatory response may contribute to better tailoring of bone substitute materials.

Based on the results of this study and the available literature, it is evident that ectopic bone formation is highly influenced by the chosen animal model, i.e. subcutaneous vs. intramuscular implantation sites, species, for example, goat vs. monkey, dog, mini-pig and sheep, as well as morphology, i.e. porous granules vs. solid structures such as cylindrical or disc-shaped materials. The granular form seems to be an influential parameter to a higher extent than previously assumed. These differences might be the reason why the implantation of the NB-granules led to ectopic bone formation in the subcutaneous and the muscle tissue of mini-pigs [12], while no osteoinductive capacity was observed when the same granules were implanted into caprine muscle.

These findings notwithstanding, our group and other groups have been able to show appreciable success with the clinical application of both bone substitute materials used here [13,14,40-42]. Despite their potential lack of *de novo* bone formation in an ectopic large animal model, they are still very relevant as viable alternatives to autografts as the search for the “ideal” bone substitute continues. The present study demonstrates that success nor failure of ectopic bone formation induced by biomaterials should not be over interpreted. The primary focus should be placed on the clinical outcome e.g. implantation and on the adaptation of the applied materials to the patient’s individual needs.

## Conclusion

The present study showed that ß-TCP granules induced more TRAP-positive multinucleated giant cell inflammatory reaction compared to NB granules. Consequently, the former underwent faster degradation. It has to be elucidated, whether the detected multinucleated giant cells within the implantation bed of the used materials are foreign body giant cells or osteoclasts. The former are involved in foreign body reactions, while the latter are known to induce the differentiation of mesenchymal stem cells into osteoblasts. The understanding of the molecular mechanism of the multinucleated giant cells involved in calcium phosphate ceramic degradation is of high relevance, especially since this knowledge might help to understand the interaction of inflammation and osteoinduction. Neither osteoblasts nor newly formed bone were detected in any of the implantation beds of the two materials at any time point during the study. The lack of osteoinduction observed here especially in the case of NB might be a result of its application form, i.e. granules and the animal model used. Osteoinduction was previously reported in goat muscle tissue related to cylindrical or disc-shaped calcium phosphate ceramics and not granules. On the other hand it has been documented that osteoinduction occurred for NB in granular form in the muscle tissue of mini-pigs. Accordingly, the question arises which material characteristics and which micro-environment are required to induce osteoinduction in calcium phosphate ceramic granules. It should however be noted that the lack of osteoinductivity in ectopic tissue in an animal model does not necessarily predict the potential of these materials to induce *de novo* bone formation in humans. Indeed clinically, the two materials investigated in this study have proven to be suitable for human bone regeneration.

## Competing interests

Hereby we confirm that this article is not reviewing by other journals. The authors declare that they have no competing of interests.

## Authors’ contributions

SG, SEU, MB and OK carried out the in vivo experiments, histological analyses as well as the histological imaging. SG, SEU, MB, IW, OK, RAS and CJK were involved in writing of the manuscript.
